# Identification of Estrogen-Responsive Proteins in Mouse Seminal Vesicles Through Mass Spectrometry-Based Proteomics

**DOI:** 10.3390/ph17111508

**Published:** 2024-11-09

**Authors:** Ammar Kapic, Khadiza Zaman, Vien Nguyen, Katalin Prokai-Tatrai, Laszlo Prokai

**Affiliations:** Department of Pharmacology and Neuroscience, University of North Texas Health Science Center, Fort Worth, TX 76107, USA; ammarkapic@my.unthsc.edu (A.K.); khadiza.zaman@unthsc.edu (K.Z.); vien.nguyen@unthsc.edu (V.N.); katalin.prokai@unthsc.edu (K.P.-T.)

**Keywords:** 17β-estradiol, male reproductive system, mouse seminal vesicle, therapeutic effects, endocrine disruption, proteomics, bioinformatics, biomarker discovery

## Abstract

**Background:** Although estrogenic compounds promise therapeutic potential in treating various conditions, concerns regarding their endocrine-disrupting effects have been raised. Current methodologies for screening estrogenicity in rodent models are limited to the female-specific uterotrophic bioassay. Studies have reported enlargement of the seminal vesicles in orchiectomized males treated with estrogens. However, identifying estrogenicity strictly through changes in wet weights is uninformative regarding the molecular mechanisms of these agents. Therefore, protein-based biomarkers can complement and improve the sensitivity of weight-based assessments. To this end, we present a discovery-driven proteomic analysis of 17β-estradiol’s effects on the seminal vesicles. **Methods:** We treated orchidectomized mice with the hormone for five days and used the vehicle-treated group as a control. Seminal vesicles were analyzed by shotgun approach using data-dependent nanoflow liquid chromatography–tandem mass spectrometry and label-free quantification. Proteins found to be differentially expressed between the two groups were processed through a bioinformatics pipeline focusing on pathway analyses and assembly of protein interaction networks. **Results:** Out of 668 identified proteins that passed rigorous validation criteria, 133 were regulated significantly by 17β-estradiol. Ingenuity Pathway Analysis^®^ linked them to several hormone-affected pathways, including those associated with immune function such as neutrophil degranulation. The altered protein interaction networks were also related to functions including endocrine disruption, abnormal metabolism, and therapeutic effects. **Conclusions:** We identified several potential biomarkers for estrogenicity in mouse seminal vesicles, many of them not previously linked with exogenous 17β-estradiol exposure.

## 1. Introduction

Estrogens are primarily associated with female reproductive health. However, they also regulate various physiological functions in both men and women in various tissues, including the central nervous and immune systems, as well as bone [1,2,3]. In mammals, 17β-estradiol (E2) is the primary estrogen, which can activate several estrogen receptors (ERs), including the nuclear estrogen receptors α (ESR1) and β (ESR2), and the G-coupled protein estrogen receptor (GPER), leading to numerous downstream genomic effects [4]. ESR1 and ESR2 act as transcription factors in the nucleus. However, they also can directly regulate signaling pathways such as the phosphoinositide 3-kinases/protein kinase B (PI3K/AKT) axis [4,5]. Conversely, GPER is restricted to the cell membrane and the endoplasmic reticulum, and its activation leads to a cascade of secondary messengers, modulating other signaling pathways [6]. Therefore, estrogens and ER agonists can potentially possess complex pleiotropic effects in various tissues.

The role of estrogens in male health is overshadowed by that of testosterone (T), which is considered the essential male sex hormone. However, induced cessation of androgen synthesis, as in androgen deprivation therapy (ADT), leads to menopause-like symptoms. These include metabolic syndrome, cognitive impairment, and osteoporosis due to lack of a sufficient level of circulating E2. The latter is formed from T by the enzyme aromatase [7,8]. Therefore, conditions associated with ADT-triggered estrogen deprivation may be remedied by exogenous supplementation of the missing hormone [9]. Recently, several small clinical studies utilized E2 delivered systemically by transdermal patches to ADT patients to alleviate the symptoms associated with estrogen deprivation. It has been reported that increased bone mineral density, as well as improved serum lipid levels and insulin sensitivity were achieved after 1 month of treatment [10,11]. Other studies using oral estrogens have shown reduced climacteric symptoms associated with ADT [12].

On the other hand, like with hormone replacement in menopausal women, the adverse side effects of elevated E2 serum levels can result in increased cancer risk, deep vein thrombosis, and feminization in males [13]. Disruption of the balance between the androgen receptors (ARs) and ERs results in aberrant signaling and dysregulation, because their activity is often antagonistic to one another. The activity of ERs is a balancing act in males, as inhibition and excessive activation lead to disease [14]. When exposed to exogenous estrogens, thereby tipping the scale toward increased ER activity, males undergo feminization and dysfunction in the male reproductive system [15,16,17,18]. Many of the studies which used transdermal and oral estrogens reported gynecomastia as the main side effect of the therapy [11,12]. Due to these caveats, the application of estrogens, estrogen-mimetics, and modulators as therapeutics (including alleviation of ADT-related symptoms) remains controversial, in part due to the lack of tissue specificity. New generations of ER modulators may possess increased specificity for ERs or tissues. Therefore, sensitive markers are required to assess peripheral estrogenicity. Additionally, there is a growing concern about exposure to endocrine-disrupting compounds (EDCs) from environmental pollutants, drug metabolites, and plastics [19]. This further highlights the need for robust screening techniques to identify and assess the impact of potentially estrogenic agents in both sexes.

Conventionally, the uterus has been used as a standard bioassay, set by the United States Environmental Protection Agency and the Organisation for Economic Cooperation and Development, to determine the estrogenicity of compounds [20]. Activation of the ERs leads to a pronounced enlargement of the uterus and increased wet weight due to water retention and endometrial growth which can be quantified without requiring advanced molecular techniques [21]. Typically, suspected estrogenic compounds are injected into immature or ovariectomized female laboratory rodents with low endogenous E2 production [20,22]. Therefore, any statistically significant enlargement of the uterus is due to the estrogenic activity of the tested agent. However, the primary limitation of this bioassay is the lack of complementary molecular markers and a male equivalent. The use of uterine weight as the sole end point may also mask weak estrogenic compounds where treatment scheduling may not allow enough time for statistically significant uterus growth, thus limiting the identification of potential EDCs. Additionally, no recognized analogous tissues have been used in male models thus far. Current standards for research recommend including both male and female groups to assess sex differences in response to a treatment or condition, and as of now, there is no male equivalent.

Several studies have observed the enlargement of seminal vesicles (SVs) in orchiectomized (ORDX) rodents exposed to estrogens. The SVs of ORDX males are usually significantly smaller than those of gonad-intact males. However, radiolabeled 5-iodo-2′-deoxyuridine uptake revealed stimulated proliferation of SVs in ORDX mice after E2 exposure [23]. Furthermore, ORDX rodents treated with the hormone had significantly larger organ weights for the SVs than the untreated groups [24,25,26]. While the SVs’ weight noticeably increased in the ORDX rodents after estrogen exposure, the growth was less drastic than that of the uterus. Like with the rodent uterotrophic bioassay, screening strictly by the wet weight of the SVs could obscure the identification of weakly estrogenic or ER-selective compounds if the mass difference is considered statistically not significant. In addition, tissue weight as a sole assay end point is ambiguous regarding the underlying molecular mechanisms of hormone action. To address this limitation, we recently published a protein-based complement to the classical rodent uterotrophic assay [27]. Utilizing label-free quantification (LFQ) and targeted proteomics, we identified and confirmed differentially expressed proteins (DEPs) as potential ER-responsive biomarkers for exogenous estrogen exposure. We therefore reasoned that screening for estrogenicity through measuring the SVs’ wet weight can also be complemented by the protein-based biomarkers approach. To this end, we report on a discovery-driven proteomic analysis of ER-driven effects on the SVs for the first time to identify DEPs for screening estrogenic compounds in males. 

## 2. Results

### 2.1. Discovery-Driven Seminal Vesicle Proteomics

Experimental samples were collected from ORDX CD-1 mice injected subcutaneously (s.c.) once daily (q.d.) for five days with E2, with the control animals having received s.c. injections of the corn oil vehicle. As expected, the SV wet weights differed significantly between the two groups (28.7 ± 3.4 mg versus 12.3 ± 1.6 mg for the E2 and vehicle treatments, respectively, as shown in Figure S1). When the raw data acquired by data-dependent (DDA) liquid chromatography–tandem mass spectrometry (LC–MS/MS) were processed with the MSFragger protein database search algorithm [28], we identified close to 700 proteins in the SV samples with Scaffold using rigorous validation criteria (Table S1). LFQ between the E2 and control groups through Scaffold revealed 133 DEPs with a fold change of ≥2. Among these proteins, 32 were upregulated, and 101 were downregulated because of E2 exposure (Table S2).

IPA^®^ associated these DEPs with both dysregulations and beneficial triggering of signaling cascades, which are involved in several biological processes and pathways (Table S3). We identified impairment of several immune-related functions, including neutrophil degranulation, gene and protein expression by Janus kinase/signal transducer and activator of transcription (JAK-STAT) signaling after interleukin-12 (IL-12) stimulation, and granzyme A signaling among the 10 most significantly enriched pathways after E2 treatment (Table 1). 

Another significant set of dysregulated pathways was associated with blood clotting, which included the response to elevated platelet cytosolic calcium ions, dissolution of fibrin clots, and metabolism of angiotensinogen to angiotensin. Additionally, the apoptotic execution phase, glutamine and glutamate metabolism, and glutathione redox reactions pathways are also shown in Table 1.

**Figure 1:**
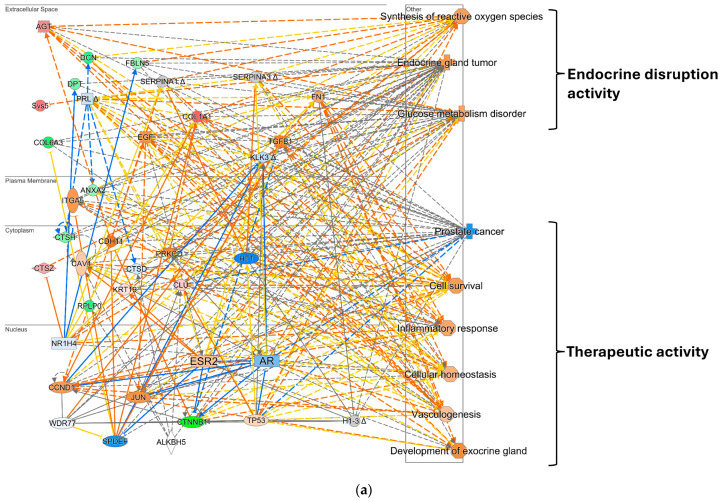

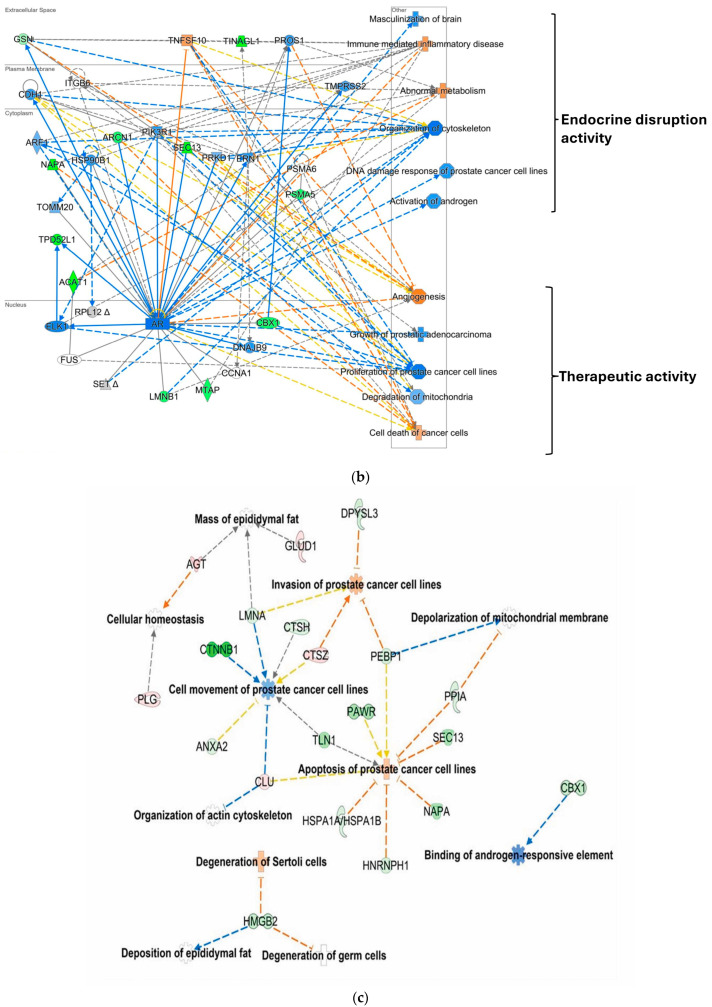
IPA^®^-generated protein interaction networks. (**a**) The top network representing cellular development, growth, proliferation, and movement. (**b**) Another network representing cell death and survival, cellular development, cellular growth, proliferation, and mapping AR-associated protein interactions. (**c**) The disease and function network, depicting specific proteins and their relationship with the development of reproductive growth and disease. Red and green colors depict quantified DEPs with increased or decreased measurements, respectively. Blue and orange depict predicted activation or suppression of the process using the MAP tool, respectively. Yellow lines indicate that the finding is inconsistent with the state of the downstream molecule. The intensity of the colors represents fold change or the Z score for the prediction. Solid and dashed lines represent direct and indirect interactions, respectively.

IPA^®^ analyses of the DEPs also revealed pathways associated with molecular and cellular functions such as cellular assembly, organization, cell death, and survival (Table 2). The biological pathways reflecting E2-triggered changes in reproductive system development, connective tissue, muscle function, as well as organ and tissue development are shown in Table 3. IPA^®^ also built 11 protein interaction networks (Tables S4 and S5 and Figure 1a,b and Figures S2–S4). The top network represented cellular development, growth proliferation, and movement, as displayed in Figure 1a. Relying on IPA^®^’s molecular activation predictor (MAP) tool, significant activation of several nuclear receptors, including ESR2 and transcription factor Jun (JUN), was predicted, while ARs were predicted to be suppressed.

While ESR2 was not in the list of submitted DEPs, its predicted activation via the MAP validated that the SVs were indeed affected by the systemic administration of E2. The relationship between ESR and AR signaling can be antagonistic; in this network, the AR is predicted to be suppressed. Further evidence of repressed AR activity is that the MAP predicted the suppression of serine protease kallikrein 3 (KLK3), also known as prostate-specific antigen. KLK3 is commonly used as a marker for AR activity and for monitoring the progression of prostate cancer [29]. Therefore, these changes in protein expression were congruent with ESR2 opposing AR-mediated functions. The top network’s associated canonical diseases and functions revealed phenotypes of both endocrine dysfunction and therapeutic effects (Figure 1a). Potentially deleterious predicted functions include glucose metabolic disorders, increased synthesis of reactive oxygen species (ROS), and the promotion of endocrine-associated cancers, which were associated with the expression pattern of this network.

Conversely, several beneficial functions were also predicted, including suppression of prostate cancer, increased cell survival, inflammatory response, cellular homeostasis, development of exocrine glands, and vasculogenesis. Many of these phenotypes from the top network (Figure 1a) are directly related to the activity of ESR2 and AR, such as reproductive system-associated cancers. Overall, these physiological functions and disease pathways revealed both the therapeutic and disruptive effects of E2 on the male reproductive system.

Another protein interaction network, shown in Figure 1b, revealed the effects of suppressing the AR-mediated pathways. Suppression of processes associated with masculinization in the brain, increased immune-mediated inflammatory disease, dysregulation in metabolism, and reduced cytoskeleton organization can eventually be considered endocrine disruption-related functions. In contrast, activation of angiogenesis, inhibition of mitochondrial degradation, and inhibition of cancer growth can be categorized as some of the beneficial impacts of E2 treatment.

Figure 1c is a depiction of functional networks created by IPA^®^ to show the regulatory effects of E2. This network displays or pinpoints proteins directly related to disease or physiological functions and their regulation pattern. This type of network can be used to select biomarker proteins in the present context. The functions associated with the network shown in Figure 1c include therapy and those associated with feminization, such as Sertoli cell degradation and reduced AR activity. The therapeutic effects are associated with the predicted suppression of prostate cancer growth and metastasis. IPA^®^ projected the association of several proteins’ downregulation, including heat shock protein families α and β (HSPA1A/HSPA1B) and peptidylprolyl isomerase A (PPIA), with prostate cancer apoptosis. E2 possibly reduced the metastatic potential of prostate cancer through reduced expression of proteins such as cathepsin H (CTSH), extracellular matrix remodeling, and β-catenin. Other functions could be associated with feminization, such as chromobox protein homolog 1 (CBX1), a mediator for the genetic activity of the AR. In our dataset, we measured CBX1 as one of the significantly suppressed DEPs. CBX1 may regulate the expression of ARs and guide ARs to genes containing the androgen-responsive element [30,31]. Thus, suppression of CBX1 may lead to reduced AR-mediated genomic activity.

Moreover, IPA^®^ linked the downregulation of high-mobility group protein 2 (HMGB2) by E2 in the SVs to three processes: maintenance of Sertoli cells in the testes, deposition of epididymal fat, and degeneration of germ cells. Nevertheless, only the degeneration of Sertoli cells was predicted to increase after E2 treatment based on HMGB2 expression. Lastly, functions related to general cellular homeostasis, epididymal fat, and mitochondrial depolarization were associated with angiotensinogen (AGT) and PPIA. However, there was no predicted direction for the pathway.

**Figure 2:**
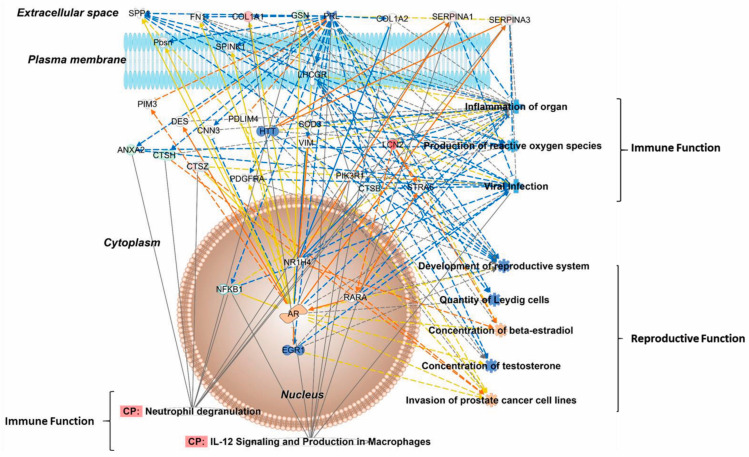
Network constructed with subset of proteins associated with neutrophil function. Blue and orange colors represent proteins’ predicted activation and suppression, respectively, for the MAP tool by IPA^®^. Red and green colors denote proteins with measured increases and decreases, respectively. Yellow dashed or solid lines indicate inconsistent findings with the downstream molecule. The intensity of the colors represents fold change or the Z score for the prediction. Solid and dashed lines represent direct and indirect interactions, respectively.

The pathway with the most significant subset of E2-regulated SV proteins was related to neutrophil degranulation (*p* = 3.1 × 10^−18^, Table 1 and Table S3). IPA^®^ also associated several other dysregulated pathways with the immune system, including IL-12 and granzyme A signaling. There is little known about the response to E2 in the immune system of the male reproductive system. Therefore, we analyzed the neutrophil degranulation canonical pathway in more significant detail to reveal the regulatory effects of E2 on the SV proteins (Figure 2 and Table S6). From this network, IPA^®^ predicted the pathways involved in the suppression of inflammation in organs, reduced production of ROS, and reduced viral infection post E2 treatment. This observation potentially focuses on the antimicrobial impacts of E2. 

Additionally, there were several reproductive functions found by IPA^®^ to be affected in this subset, including suppression of T and increased E2 concentration. Dysregulation in the reproductive system was an additional prediction, with a reduction in Leydig cells among the functions. However, IPA^®^ predicted activation of the AR despite the reduced androgen concentrations (Figure 2). Nevertheless, there have been few studies depicting the reactivation of ARs under androgen deprivation. Though the molecular mechanisms are still elusive, some possible mechanisms include AR mutations, increased transcriptional coactivator proteins, or signaling cascade regulation in increasing AR response under low T levels [32]. Due to the complexities associated with protein signaling and the crosstalk between hormone receptors, these functions may not be appropriately attributed to ERs in the male reproductive system.

### 2.2. Bioinformatics-Based Biomarker Identification

**Figure 3:**
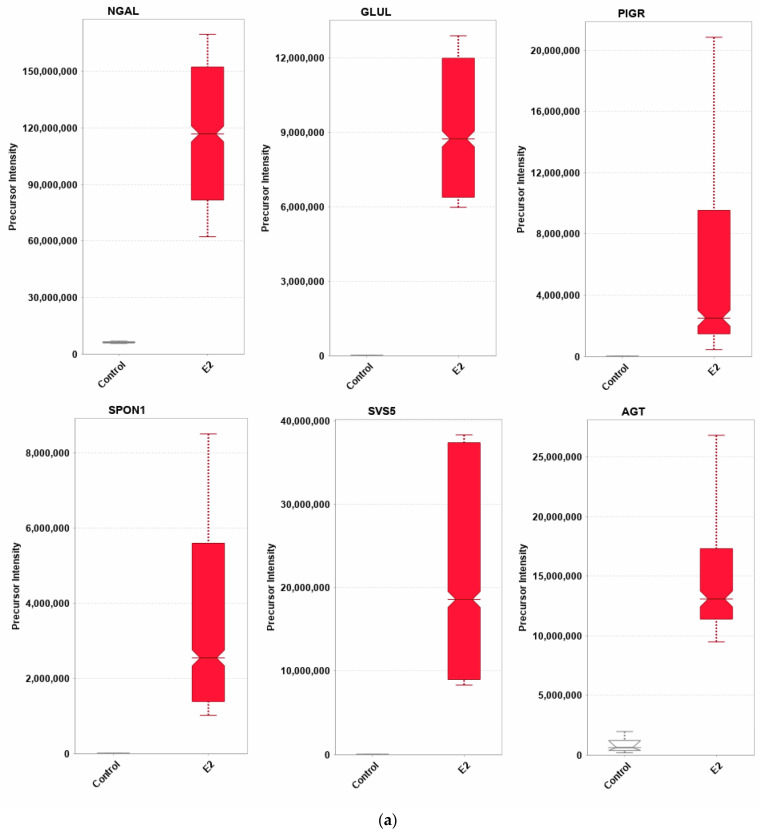

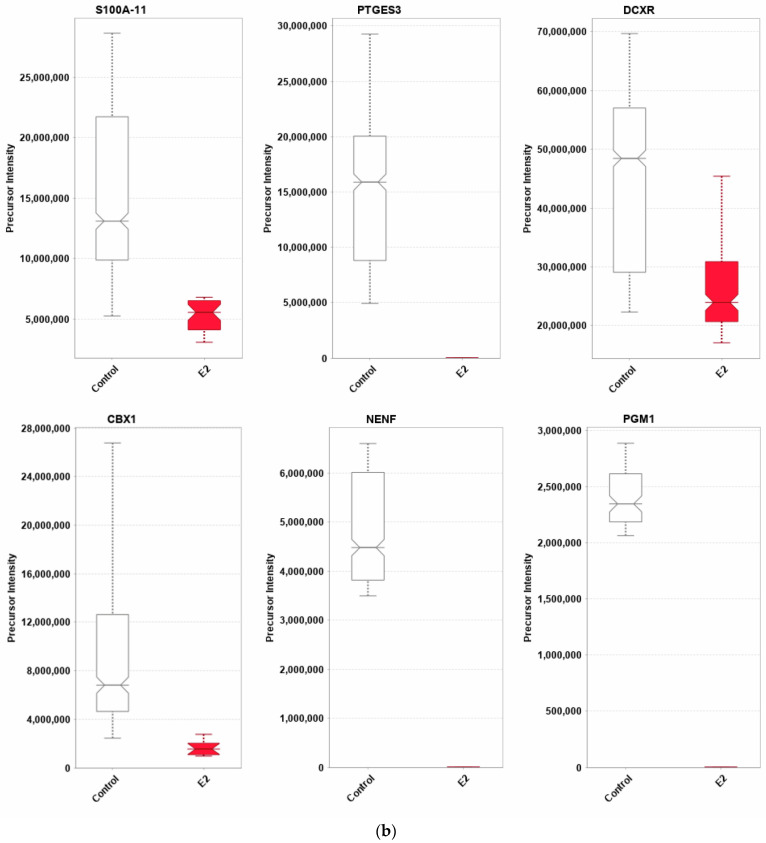
Fold changes of the 12 chosen representative SV proteins significantly affected by E2 treatment. (**a**) The panel significantly upregulated proteins in response to E2 treatment, namely neutrophil gelatinase-associated lipocalin (NGAL), glutamine synthase (GLUL), polymeric immunoglobulin receptor (PIGR), spondin-1 (SPON1), seminal vesicle secretory protein (SVS5), and angiotensinogen (AGT). (**b**) Significantly downregulated proteins in response to E2 treatment: protein S100A11, prostaglandin-synthase 3 (PTGES3), L-xylulose reductase (DCXR), chromobox 1 (CBX1), neudisin-1 (NENF), and phosphoglucomutase-1 (PGM1). Fold changes were calculated by Scaffold from tryptic peptide precursor intensities. All proteins in the figure were significantly dysregulated (*p* < 0.05) by E2 treatment; *p* values can be found in Table 4.

Twelve DEPs profoundly affected by E2 treatments were selected as a panel of potential biomarkers for estrogenic exposure in mouse SVs (Table 4). Some of these proteins were exclusive in either the control group or the E2-treated group. The upregulated DEPs include glutamine synthetase (GLUL), AGT, spondin-1 (SPON1), seminal vesicle secretory protein 5 (SVS5), neutrophil gelatinase-associated lipocalin (NGAL), and polymeric immunoglobulin receptor (PIGR) (Figure 3a). On the other hand, the following DEPs were significantly suppressed according to their precursor intensities: neudesin (NENF), L-xylulose reductase (DCXR), phosphoglucomatse-1 (PGM-1), protein S100-A11 (S100A11), chromobox homolog protein 1 (CBX1), and prostaglandin E synthase 3 (PTGES3) (Table 4, Figure 3b). The pathway explorer tool of IPA^®^ hypothesized that E2’s effects on these biomarkers were associated with both therapeutic and endocrine-disrupting effects.

**Figure 4:**
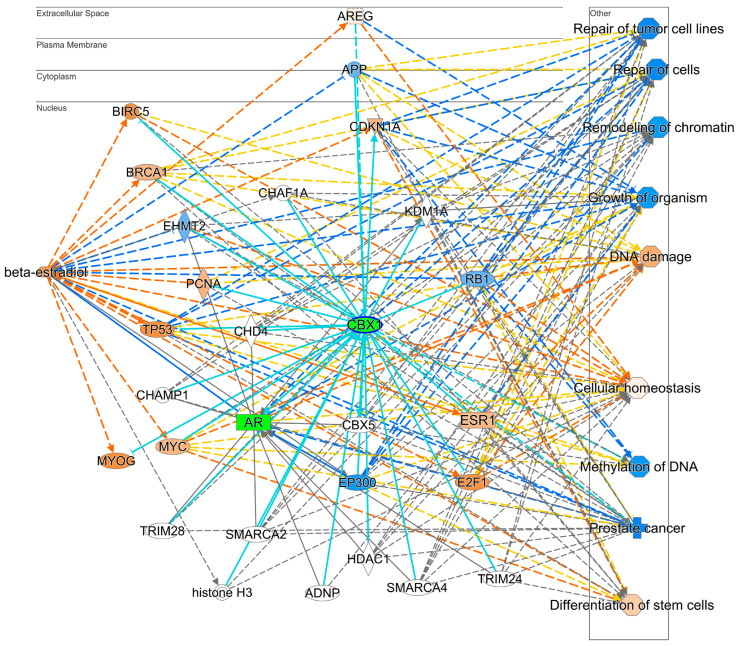
Protein interaction network and impacted biological functions generated by the hypothesis-generating pathway explorer tool depicting SPON-1 interaction with E2. No direct interaction between the ER and SPON-1 was found by IPA^®^. Blue and orange colors represent proteins’ predicted activation and suppression by the MAP tool of IPA^®^, respectively. Red and green colors denote proteins which were found to increase and decrease, respectively. Teal solid and dashed lines indicate the closest relationship pattern of SPON1 with other proteins. The intensity of the colors represents fold change or the Z score for the prediction. Solid and dashed lines represent direct and indirect interactions.

**Figure 5:**
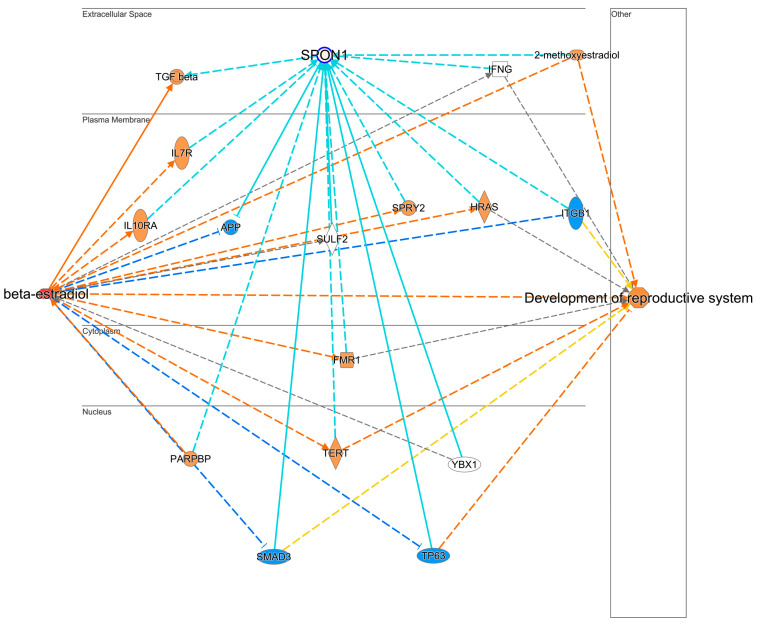
Protein interaction network and impacted biological functions generated by the hypothesis-generating pathway explorer tool. The network represents interaction between E2 and CBX1. Blue and orange colors represent proteins’ predicted activation and suppression by the MAP tool of IPA, respectively. Red and green colors denote proteins which were found to increase and decrease, respectively. Teal solid and dashed lines indicate the closest relationship pattern of CBX1 with other proteins. The intensity of the colors represents fold change or the Z score for the prediction. Solid and dashed lines represent direct and indirect interactions, respectively.

Among these biomarkers, we highlighted the previously uncharted and hypothesis-driven impacts of E2 on SPON1 and CBX1, as information is scarce regarding these proteins’ interactions with E2 and their receptors. Utilizing the pathway explorer tool in IPA^®^, we individually generated interaction networks between E2 and SPON1 (Figure 4) and CBX1 (Figure 5) to determine the potential direct or indirect relationships with E2 and ERs. Currently, no published studies contain information on the changes in the quantity of the protein itself; instead, only studies focusing on mRNA expression changes exist.

To isolate the interaction between SPON-1 and E2, the pathway explorer tool was utilized to generate the IPA^®^ network shown in Figure 4 and Table S7, which reported no direct interaction with E2 or an ER but revealed an indirect relationship through several proteins such as interleukins (IL7/10 R). Paradoxically, the MAP tool of the software predicted suppression of SPON-1. However, we reported its significant upregulation in the SV upon exposure to E2. Nevertheless, the MAP tool of the software predicted the activation of the reproductive system development function through this interaction.

In Figure 1c, the reduced expression of CBX1 was associated with suppressed activity of AR-targeted genes. In Figure 5 and Table S8, a direct link between E2 and CBX1 was not found. However, the relationship between ESR1 and CBX1 was implicated (teal lines), but there was not a predicted relationship between the two. The suppression of CBX1 is related to several phenotypes mentioned in previous figures. Prostate cancer-associated functions were predicted to be suppressed, including repair of tumor cells, chromatin remodeling, cellular homeostasis, and reduced methylation of DNA. Therefore, evidence at the protein level suggests that ESR1 negatively regulates CBX1.

## 3. Discussion

Estrogens regulate various molecular pathways and functions by modulating the ERs. The beneficial broad-spectrum effects of estrogens, such as E2 and ER mimetics, have renewed their interest as potential therapies to address multifactorial diseases, such as those associated with menopause and ADT [7,13]. However, the lack of specificity of these compounds often leads to severe peripheral side effects, including deep vein thrombosis, cancers, and feminization in males [55]. For this reason, accidental environmental exposure to estrogens and estrogen-mimicking compounds has become a growing concern [19]. Therefore, to assess for peripheral side effects and identify estrogenic compounds, the uterotrophic bioassay has been used as a quick and straightforward method to determine these effects in a rodent animal model [20]. However, the application of a uterotrophic bioassay is limited in context of, for example, ADT and estrogenic endocrine disruption in males because there is no recognized analogous male equivalent to assess these effects. Therefore, identification of sex-specific effects or responses to estrogenic compounds or their delivery have been disregarded. Secondly, lacking additional biomarkers may leave out critical information, such as male-specific ER targets, or reveal the effects of weakly estrogenic compounds in males. Previous studies have shown estrogen-dependent growth of the SV, making it an estrogen-sensitive tissue potentially analogous to the uterus [24,25]. However, this growth is not as stark as that of the uterus, as seen in Figure S1. Therefore, our study sought to evaluate the response in the proteome of the mouse SVs and is the first to report potential estrogen-responsive protein markers in a male reproductive tissue.

Based on the expression pattern of the submitted DEPs, ESR2 was predicted to be activated while the AR was suppressed (Figure 1a). ESR2 was not found among the identified proteins due to its low abundance. Nevertheless, the emergence of this protein interaction indirectly validates that the protein expression changes observed were due to the E2 treatment. Specifically, ESR2 was predicted to be activated instead of the other ERs, suggesting that this particular ER may play a prominent role in regulating these downstream effects. However, this will need to be validated using selective estrogen receptor agonists and antagonists to isolate which ERs contribute to the changes in protein expression in the networks. In our study, only E2 was utilized, which lacked ER specificity. Future studies could expand these experiments to isolate ER isoform-specific responses, which can give valuable insight when screening for suspected estrogenic compounds. 

The activity of the ERs and ARs are antagonistic in both their response and their synthesis. In males with normal hormone levels, exposure to estrogens leads to feminization due to the balance of ER and AR signaling shifting toward ER-mediated pathways. In our protein network, the AR was predicted to be suppressed by E2 downregulating some AR-responsive proteins such as the classic KLK3 or prostate-specific antigen, a marker for AR activity [56]. Interestingly, the ER-mediated pathway predicted that the transcription factor JUN would be activated by E2. JUN is a crucial cell cycle regulator, and increased activity directly promotes proliferation [57]. The hormone T was shown to inhibit the proliferation and oxidative phosphorylation in the SV epithelial gland cells, which was reversed using flutamide, an AR inhibitor [58]. In this context, the AR promoted the formation of oleic acid for the seminal fluid, reducing the utilization of glucose for energy and proliferation. In Figure 1b, reduced mitochondrial degradation is a predicted function, suggesting that the reproductive function of producing biomolecules for seminal fluid may be inhibited after E2-mediated suppression of the AR. Additionally, exocrine development, which includes the SVs, was linked to E2 exposure by IPA^®^, as shown in Figure 1a.

In our animal model, the mice were already deprived of sex hormones. However, the antagonistic relationship between the ER and AR can also be seen in their predicted activation in Figure 1a,b. CBX1, which is involved in gene silencing through methylation, is a coactivator for androgen response element (ARE) genes [30]. The expression of CBX1 is elevated in prostate cancer and directly promotes the binding of the AR to the ARE [30]. Thus, one of the mechanisms by which E2 treatment may contribute to restricting PCa growth by suppressing ARE genes is shown in Figure 1c.

Treating ADT patients with E2 may elicit similar effects by promoting cellular function while suppressing the growth of AR-driven cancers such as renal and prostate cancer, as shown in Figure 1, bolstering their immune systems, and promoting cellular homeostatic mechanisms which could eventually lead to symptoms similar to menopause. However, some potential outcomes, such as increased ROS production and glucose metabolism issues, were predicted to be related to the endocrine disruption. The latter is associated with high concentrations of estrogens in males, mainly promoting exocrine cancers. However, the effects of estrogens are biphasic, and at a lower dosage, there could be less metabolic dysregulation [59]. Patients in the PATCH clinical trial showed improved fasting cholesterol and glucose levels six months and one year after ADT [11].

Many DEPs were associated with aspects of the immune system, including neutrophil degranulation, STAT-JAK gene expression, granzyme A, and glutamate and glutamine metabolism potentially related to nitric oxide production [60] (Table 1). Few studies have explored the immune system’s response to estrogens in the male reproductive system. Overall, inflammation was predicted to be upregulated in the cellular growth and development network and the network representing AR-associated proteins (Figure 1a,b). T is recognized as an immunosuppressant in the male reproductive system to prevent autoimmunity toward sperm cells [61]. In contrast, estrogens appear to bolster the immune response [62]. The treatment with E2 in this ORDX model for androgen deprivation was predicted to regulate inflammatory effects in some contexts, according to the protein networks and pathways in mouse SVs.

The most prominently dysregulated pathway was associated with neutrophil degranulation, a process related to the innate immune system (Table 1). Neutrophils are leukocytes found in abundance throughout the body and often regulate inflammation by releasing cytokines at bacterial or fungal infection sites [63]. However, apoptosis of neutrophils can mediate anti-inflammatory phenotypes in other cell types to promote wound healing. E2 may mediate neutrophil activity dose-dependently, because high dosages prevent the degranulation of specific proteins while physiological levels may promote their release [64,65]. Reduced degranulation would indicate some anti-inflammatory regulatory effects of E2 on the innate immune system. According to the predictive network related to neutrophil degranulation (Figure 2), prolactin (PRL) expression was predicted to be inhibited. PRL is associated with inflammation by interfering with regulatory T cells (T-Regs) and promoting pro-inflammatory macrophages [66,67]. Lipocalin-2 (LCN2) is another central mediator of inflammation, serving dual roles in both the active and innate immune systems. In macrophages, LCN2 promotes the anti-inflammatory M2 phenotype and T-Reg cell proliferation [68,69]. However, LCN2 is usually released during bacterial infection to sequester iron and is recognized as a marker for kidney damage [70]. According to this population of DEPs, LCN2 could play an immunosuppressive role in some immune cells.

Interestingly, the changes in protein expression in Figure 2 are predicted to be regulated by ARs instead of ERs. However, numerous yellow lines suggest unconfirmed interactions or are contractionary to the literature. This phenomenon could be attributed to the fact that these functions are either shared between the ER and AR or are regulated by the ER in a compensatory mechanism. Androgens are reported to be generally immunosuppressive primarily to protect sperm production, while estrogens may bolster immune responses in some contexts [71]. T can be locally converted into E2 by the action of aromatase. Therefore, it is difficult to isolate the activity of the AR. ORDX mice have deficient T levels. However, the ER may compensate for these normally AR-related functions in this hormone-deprived state in the context of this protein interaction network.

The other significantly dysregulated immune-related functions included serine-protease, granzyme A (GZMA), signaling, and downstream JAK-STAT signaling in response to interleukin 12 (IL-12), which are involved in innate and active immune system functions (Table 1). Natural killer (NK) and cytotoxic T -cells (CTLs) can induce apoptosis in cells expressing pro-death proteins on their surfaces, often as a result of pathogen infection or those with tumorigenic potential [72]. Expression of IL-12 can promote the proliferation of NK and CTLs and bolster their GZMA production, which can influence other immune cells or induce apoptosis [73]. However, the apoptotic execution phase was predicted to be inhibited. Hormone deprivation is associated with reduced immune response. Therefore, E2 treatment may have ameliorated the function of the immune system instead of leading to pro-inflammatory effects [74].

Among the reported DEPs, we focused on several proteins with the potential to serve as biomarkers for estrogenic activity in mouse SVs (Table 4 and Figure 3). These proteins were significantly dysregulated, with a fold change of at least two. Our panel of upregulated DEPs could serve as positive biomarkers for E2 exposure. As previously mentioned, the glycoprotein LCN2 was involved in the network in Figure 2 and modulated the immune system. Under normal physiological conditions, the expression of LCN2 is low [75]. However, LCN2 can sequester iron during bacterial infections to reduce the proliferation of pathogenic bacteria and initiate wound healing [76]. Elevated expression is typically only found in cancers, while mRNA in the male reproductive system is undetected in the SVs [75,77]. Our study is potentially the first to detect the presence of LCN2 protein in mouse SVs. Lastly, the LCN2 gene possesses an ERE and has been shown to be responsive to estrogens [78]. In non-gonadectomized murine reproductive tissues, knockouts of ESR1 expressed elevated LCN2. In contrast, the liver, kidney, and lung ESR1 knockouts had reduced LCN2 expression [79]. Therefore, ESR1 may regulate its expression. However, in the case of gonadectomized males, E2 may significantly promote its expression, making it an excellent positive biomarker for estrogen exposure.

GLUL, a ligase which catalyzes glutamine synthesis using glutamate and ammonia through an ATP-dependent reaction, was also significantly upregulated [80]. The generation of glutamine is vital for producing other macromolecules, including nucleotides and proteins, regeneration of glutathione, and regulating nitrogen levels in the body [81]. Elevated expression of GLUL in response to estrogen has been reported [82,83]. Exposure to orally delivered diethylstilbestrol (DES), a known estrogenic endocrine-disrupting compound, elevated the expression of GLUL in the testes, which was suggested by the authors to be a stress response for glutathione regeneration [83]. The introduction of E2 to a sex hormone-deprived SV may have induced a strong signal for growth, resulting in an elevated need for biomolecules and thus the need for glutamine to synthesize nucleotides and proteins [80]. Additionally, future studies could utilize glutamine concentrations to validate GLUL expression regarding estrogenic compound exposure similar to that in the study conducted earlier [37].

Expression of the transmembrane receptor PIGR is predominately limited to the epithelial layers, where it transports antibodies such as IgA and IgM to the mucosa [84]. While PIGR expression has been found along the reproductive tract, our study is the first to provide evidence for its expression in the SVs. As reflected in our data, ERs and ARs can upregulate PIGR expression, as E2 treatment significantly upregulated protein expression [85]. Because PIGR is involved in the pathogenic response, elevated expression indicates that the immune system was bolstered in response to E2. Lastly, expression of PIGR was found only in the seminal plasma of patients with varicocele infertility [39].

Little is known about SPON1, an adhesion protein involved in neuronal development. Only a few studies about its expression and function have been published in the past 30 years. SPON1 is secreted by the floor plate, a temporary structure beneath the neural tube during embryonic development, and is believed to regulate axon guidance [86]. However, elevated expression has been reported in malignant ovarian cancers. In endometrial cancers, increased SPON1-induced apoptosis [87,88,89]. There is no direct link between E2 and SPON1 expression, as shown in Figure 4. According to IPA^®^, SPON1 was predicted to be repressed after E2 treatment. However, we observed elevated SPON1 levels in our datasets. A single publication utilizing the E2 metabolite 2-methoxyestradiol (2-ME), which binds poorly to the nuclear ER, showed increased expression of SPON1 in endometrial cancer cell lines [89]. As 2-ME can bind to GPER, whether this expression is tied to inhibition of the nuclear ER or through GPER is unknown. However, this 2-ME may be categorized as an ER antagonist. Therefore, based on the knowledge base of IPA^®^, it was predicted to be inhibited. Our study is the first to report its expression in the SVs in response to E2.

Another understudied protein, SVS5, and other SVS proteins have historically been used as a marker for AR activity in androgen-responsive male reproductive organs [90]. These proteins are transcribed from the semenogelin 1 and 2 genes and are involved in forming a copulatory plug and encapsulating sperm during ejaculation [91]. As a marker for AR activity, SVS4 (another member of the SVS family) was suppressed in ORDX animals and, when treated with T, resulted in elevated mRNA [92]. Other studies using gonad-intact animals have shown suppression of SVS5 expression after treatment with either estrogens or estrogenic compounds such as bisphenol A (BPA). Exposure to higher concentrations of ethinylestradiol, BPA, and genistein suppressed the expression of SVS5 mRNA in gonad-intact males. For example, medium dosages of BPA, ranging from 0.02 to 50 mg/kg, led to elevated expression of SVS5 mRNA [93]. However, the SV smooth muscle expression of SVS5 and other SVS proteins did not change in AR-knockout mice [94]. In our study, we saw an elevated expression of SVS5 after E2 treatment. Therefore, there is a possibility that E2 may mediate the expression of SVS5 in a compensatory mechanism under sex hormone deprivation or may regulate its protein expression. Future studies are needed to validate these results.

Lastly, our results indicated the upregulation of AGT, the precursor regulator of the renin-angiotensin system (RAS). Classically, AGT regulates fluid retention in the body, and when cleaved by angiotensin-converting enzyme (ACE), the processed proteins initiate their own signaling cascades and regulatory functions. Seminal AGT levels were found to be correlated with infertility and poor sperm motility [39,44]. Interestingly, the proteomic analysis of seminal plasma in infertile men have suggested that dysregulation of Y-box binding protein (YBX-1) in the seminal plasma may lead to elevated AGT concentrations. In Figure 4, YBX1 was linked to SPON1. However, IPA did not predict its specific activity. Alternatively, estrogens and sex hormone deprivation suppress the activity of ACE in males by promoting ACE2 expression, which antagonizes ACE activity [95]. On the other hand, ACE expression in our dataset was elevated, and ACE2 was not among the DEPs. Estrogen replacement therapy has been shown to reduce ACE activity in menopausal women. However, no study has investigated AGT or ACE in the context of sex hormone-deprived animal models [96]. In our dataset, AGT was upregulated, and it may serve as a biomarker, as it was found in the seminal plasma in addition to the SVs.

Our panel of suppressed DEPs can serve as negative biomarkers to show the repressive effects of the ER, mediated by ARs and inflammation. S100A11 regulates the activity of cell cycle-associated proteins such as RAD54B, EGF, and nucleolin [45]. Typically, S100A11 is heavily expressed in both male and female reproductive systems and regulates immune tolerance in those tissues. However, only in females has the role of S100A11 been explored [97]. The effects of estrogens on S100A11 expression are only limited to transcription data, and according to several studies, E2 reduced its expression [98]. However, in our dataset, at least in the context of the SV, estrogens promoted S100A11 protein expression.

Several different prostaglandins are found in the seminal plasma. PTGES3, also known as p23, produces prostaglandin E2 (PGE2), a mediator of inflammation from prostaglandin endoperoxide H2. PGE2 has immunosuppressive effects in seminal plasma [99]. In breast and cervical cancers, E2 was found to upregulate the expression of PTGES3, potentially promoting an immunosuppressive environment [100,101]. However, exposure of neonatal males to estrogens such as BPA, genistein, and DES did not affect PTGES3 mRNA expression in the testis [102]. Studies suggest that PTGES3 regulates AR- and ER-mediated transcription, but no studies have shown that E2 suppressed the protein expression of PTGES3, as shown in our dataset [103,104].

DCXR mediates the conversion of L-xylulose into the sugar alcohol xylitol and, more importantly, metabolizes toxic α-dicarbonyl compounds in the reproductive system, and it is also found on the surface of sperm cells [51]. Protein expression of DCXR is typically high in the male reproductive system and the seminal plasma. DXCR activity was shown to protect sperm cells from the toxic glyoxal, reducing glycation in the sperm cells [105]. Response to estrogens may be sex-linked, as mRNA levels have been shown to increase in response to E2 in breast cancer, while exposure to BPA in placental explants resulted in decreased protein expression [82,106]. Likewise, we saw significant suppression of the protein in our dataset.

CBX1, a histone H3 Lysine 9 methyl reader and nuclear lamina protein involved in gene silencing, is heavily implicated in promoting several cancers, such as prostate cancer [30]. One of the functions of CBX1 is regulation of the genomic-mediated pathways of the AR, as it acts as a coactivator [107]. In Figure 1c, expression was directly related to AR-mediated activity. We measured reduced CBX1 expression in our dataset. This suggests that the expression of AR-mediated pathways may be repressed. Elevated expression and activity have been found in metastatic prostate cancers, where it may promote the activity of ARs [108]. Suppression of CBX1 is directly associated with ESR1 activation according to the MAP tool, as shown in Figure 5. Increased breast cancer susceptibility gene 1 (BRCA1) directly results from the negative feedback loop which regulates ESR1, as BRCA1 inhibits the expression and downstream activity of ESR1 [109]. However, the DNA binding function of BRCA1 requires CBX1. Therefore, repressed CBX1 could prevent BRCA from playing its inhibitory role [109,110].

Studies relating to NENF are primarily relegated to its function in the brain, such as regulating anxiety and food intake [111]. NENF is expressed in other tissues, including the male reproductive system. While its function remains unclear, it may play a role in developing the male reproductive system [112]. E2 treatment affects the expression of NENF mRNA differently; in rat hepatic cells it is decreased, but in breast cancer cell lines it is elevated [53,113]. Lastly, PGM1 was significantly downregulated in response to E2 treatment. PGM-1 regulates glucose metabolism by moving the phosphate position on glucose to prepare for either glycogen synthesis or glycolysis [114]. Reduced expression could reduce glycogen-mediated metabolic activity. However, the activity of PGM1 is regulated by kinases such as PAK1 [115].

With the development of new ER modulators, more robust screening techniques are required, especially those which can include both male and female subjects. In this study, we reported several protein preclinical biomarkers, functional pathways, and protein interaction networks related to the response of E2 in the SVs of ORDX male mice. Both therapeutic and endocrine-disrupting functions were found post E2 treatment, with the protein networks suggesting increased immune system function and growth of the SVs. While our panel demonstrated the pleiotropic effects of E2 on mouse SVs, further validation, such as using targeted proteomics or other protein quantitative techniques, is required. These preclinical biomarkers could be used as a complementary set of surrogate end points alongside the changes in SV weight. Therefore, the SV can act as an analogous tissue to the uterus to ascertain the estrogenicity of compounds and be used as a marker for peripheral exposure.

## 4. Materials and Methods

### 4.1. Chemicals and Reagents

E2 and other chemicals were purchased from Millipore Sigma (St. Louis, MO, USA). Sequencing-grade trypsin was acquired from Promega (Madison, WI, USA). Chromatographic solvents were of Optima^®^ LC/MS grade and supplied by Thermo Fisher Scientific (Waltham, MA, USA). The agents used for euthanasia were purchased from Covetrus (Fort Worth, TX, USA).

### 4.2. Animal Model

ORDX male CD1 mice (25–30 g) were purchased from Charles River Laboratories (Wilmington, DE, USA). The animals were housed under standard conditions with ad libitum access to food and water. The experiments performed were outlined in the IACUC protocol IACUC-2018-004 and per the animal care guidelines of the University of North Texas Health Science Center. Mice were subcutaneously (s.c.) injected with either 100 µg/kg body weight of E2 in a corn oil vehicle (*n* = 4) or with the vehicle alone (*n* = 4) once daily (q.d.) for five days between 10:0-0 a.m. and 11:00 a.m. local time. Twenty-four hours after the last dose, the animals were euthanized by intraperitoneal injection of ketamine (80 mg/kg body weight) and xylazine (7 mg/kg body weight), followed by decapitation. An abdominal incision was then made to take out the SVs. After removing excess fat and connective tissues, each SV was blotted and immediately weighed to record the wet weight.

### 4.3. Proteomics

Individual SVs were incubated at room temperature for an hour in a lysis buffer of 8 M urea in 25 mM ammonium bicarbonate. Reduction, alkylation, trypsin digestion, and sample cleanup were performed according to our previously reported procedures [116]. The samples (1 µg/µL protein content) were reconstituted in 5% aqueous acetonitrile containing 0.1% formic acid (FA). Data-dependent LC–ESI-MS/MS was run on an LTQ Orbitrap Velos Pro mass spectrometer connected to EASY nLC-1000 systems and fitted with an EASY-Spray source (Thermo Fisher Scientific, San Jose, CA, USA). The source voltage and ion transfer tube temperature were 2.2 kV and 275 °C, respectively. Nanoflow separations were performed on a 15 cm × 75 μm i.d. EASY-Spray column packed with 3 µm PepMap C18 particles. Peptides were eluted at a 300 nL/min flow rate with a 2 h binary solvent gradient mixed with water and acetonitrile containing 0.1% formic acid [116]. During elution, a full-scan MS was acquired with a nominal mass resolution of 60000 (at *m/z* 400) in the Orbitrap, and up to 20 MS-dependent MS/MS scans were obtained in the ion trap. Each full MS/MS spectrum was acquired using collision-induced dissociation (CID) of multiple charged ions with z ≥ 2. After selecting the ion to be fragmented, dynamic exclusion was set for 60 s. Two technical repeats were analyzed for each sample.

### 4.4. Data Analysis

MS/MS spectra were searched against the UniProt mouse protein database using the Mascot search engines (version 2.6.2; Matrix Science, Boston, MA, USA) in Proteome Discoverer (version 2.4; Thermo Fisher Scientific, San Jose, CA, USA). Parent ion tolerance of 25 ppm, fragment ion mass tolerance of 0.80 Da, and one missed cleavage were set as search filters. Fixed and variable protein modification settings included carbamidomethylation of cysteine, oxidation of methionine, and acetylation at the N-termini. The search results were validated to meet decisive criteria of protein identification using the Peptide Prophet and Protein Prophet algorithms, requiring >95% and >99% probabilities, respectively, and at least two identified unique peptides for each protein using Scaffold software (version 5.3.3, Proteome Software Inc.; Portland, OR, USA) [117]. Statistical significance was determined via *t*-tests. Proteins with a fold difference of ≥2 were considered a threshold of significant impact. The identified E2-regulated proteins in the SVs were then submitted to Ingenuity Pathway Analysis^®^ (IPA^®^, QIAGEN, Redwood City, CA, USA) to derive bioinformatics annotations along with potential protein interaction networks, as well as associated biological functions and processes [27,116]. Overlaps of p values were reported from IPA^®^’s calculations using the right-tailed Fisher’s exact test. Z scores were generated for regulated functions and their predicted signaling patterns through the MAP tool built into IPA^®^.

## 5. Conclusions

Like the uterotrophic bioassay, the SVs of ORDX male mice can be used to mark for estrogen exposure. However, relying on weight changes alone may mask weakly estrogenic compounds and is uninformative regarding molecular mechanisms of the hormone’s action. In this study, we utilized discovery-driven proteomics to assess the response to E2 in mouse SVs and recognized 12 proteins which may be used as surrogate protein biomarkers for estrogenicity in males.

## Figures and Tables

**Table 1:** IPA^®^’s top canonical pathways regulated in the SVs upon E2 treatment. Positive Z score values predict activation, while negative Z scores forecast suppression. N/A represents no predictions made.

Canonical Pathways	Number of Associated Molecules	−log (*p* Value)	Z Score
Neutrophil degranulation	37	17.5	−1.732
Response to elevated platelet cytosolic Ca^2+^	10	7.83	1.265
Apoptotic execution phase	7	7.36	−2.0646
Gene and protein expression by Jak-STAT signaling pathway after IL-12 stimulation	6	6.88	0.816
Granzyme A signaling	6	5.13	2.449
Cellular response to heat stress	6	4.39	0.449
Dissolution of fibrin clot	3	4.14	N/A
Glutamate and glutamine metabolism	3	4.03	N/A
Trans-Golgi network vesicle budding	5	3.92	−1.342
Metabolism of angiotensinogen to angiotensin	3	3.69	N/A

**Table 2:** Top E2-regulated molecular and cellular functions in mouse SVs.

Molecular and Cellular Functions	Number of Associated Molecules	*p* Value of Overlap
Cellular assembly and organization	3	3.82 × 10^−2^–4.19 × 10^−5^
Cellular function and maintenance	5	3.84 × 10^−2^–4.19 × 10^−5^
Cell death and survival	9	3.21 × 10^−2^–5.61 × 10^−5^
Cellular movement	9	4.13 × 10^−3^–1.57 × 10^−3^
Cellular development	4	6.50 × 10^−3^–3.53 × 10^−3^

**Table 3:** Top E2-regulated biological pathways in mouse SVs.

Biological Pathway	Number of Associated Molecules	*p* Value of Overlap
Connective tissue development and function	5	3.84 × 10^−2^–6.50 × 10^−3^
Organ development	2	2.58 × 10^−2^–6.50 × 10^−3^
Reproductive system development and function	6	3.84 × 10^−2^–6.5 × 10^−3^
Tissue development	3	2.58 × 10^−2^–6.5 × 10^−3^

**Table 4:** The panel of potential SV biomarkers for estrogenic exposure with their recognized E2-associated functions.

Gene	Protein	Fold Change Upon E2 Exposure	*p* *	Function
NGAL/LCN2	Neutrophil gelatinase-associated lipocalin	18	0.01	Acute inflammation marker [33]. Potential marker for acute kidney injury and metabolic disorders [34,35].
GLUL	Glutamine synthetase	12	0.011	Mediates the synthesis of glutamine [36]. Expression is tied to estrogen receptors in glial cells [37].
PIGR	Polymeric immunoglobulin receptor	9.5	0.032	Transmembrane transporter found on mucosal surfaces. Transports IgA and IgM. Maintains mucosal immune functions [38]. Increased expression in males with varicoceles, a condition which may lead to male infertility [39].
SPON1	Spondin-1	8.3	0.001	mRNA expression was found in various tissues. However, protein expression was not detected [40]. Function unknown in the male reproductive system [41].
SVS5	Seminal vesicle secretory protein 5	7.5	0.016	Processed by prostate-specific antigen, involved in promoting sperm motility. Elevated levels may promote male infertility [42].
AGT	Angiotensinogen	6.5	0.016	Involved in the regulation of the renin-angiotensin system [43]. Seminal AGT may be involved in regulating sperm motility [44].
S100A11	Protein S100-A11	−2	0.045	Calcium-binding protein is involved in various regulatory functions [45]. Considered a marker for inflammation. Upregulated in colon and prostate cancers [46,47].
PTGES3	Prostaglandin E synthase 3	−2.5	0.028	Promotes the function of steroid receptors in the immune system, and mRNA expression may be reduced after exposure to bisphenol A [48,49].
DCXR	L-Xylulose reductase	−2.5	0.004	Short-chain reductase metabolizes xylulose, quinone, and diacetyl with other potentially unknown functions [50]. Sperm surface protein, essential for gamete fusion and fertilization, is reduced in patients with SCI-induced male infertility [51,52].
CBX1	Chromobox protein homolog 1	−3.3	0.014	Androgen receptor cofactor and regulates the androgen receptor’s genomic activity. Upregulated in castration-resistant prostate cancer [30].
NENF	Neudesin	−5	0.008	Function in the male reproductive system has yet to be fully elucidated. Expression at the mRNA level may be reduced after ER activation but is cell-specific [53].
PGM-1	Phosphoglucomutase-1	−5	0.006	Involved in glycogen metabolism and N-glycosylation. Reduced expression in patients who have SCI-induced infertility [52,54].

* Post hoc *t*-test: E2-treated versus control.

## Data Availability

The mass spectrometry proteomics data have been deposited to the ProteomeXchange Consortium via the PRIDE [118] partner repository with dataset identifiers PXD056329 and https://doi.org/10.6019/PXD056329.

## Supplementary Materials

The following supporting information can be downloaded at https://www.mdpi.com/article/10.3390/ph17111508/s1. Figure S1: Seminal vesicle weight comparison between control and E2-treated animals. Figure S2: IPA^®^ network linked to cellular development, embryonic development, and organismal development. Figure S3: IPA^®^ network linked to cancer, cellular movement, organismal injury and abnormalities. Figure S4: IPA^®^ network linked to cellular movement, hematological system development and function, and immune cell trafficking. Table S1: List of identified and validated proteins (Scaffold 5.3.3: >95% and >99.9% probability by Peptide Prophet and Protein Prophet, respectively, with a minimum of two unique peptides) from the control and E2-treated seminal vesicles, along with their gene ontology annotations. Table S2: Differentially expressed seminal vesicle proteins (*t*-test, *p* < 0.05) mapped by IPA^®^, with fold change ≥2. Table S3: List of all regulated canonical pathways with their associated molecules generated by IPA^®^. Table S4: List of all protein interaction networks generated in IPA^®^. Table S5: List of all protein names abbreviated in Figure 1a–c with their functions. Table S6: List of all protein names abbreviated in Figure 2 with their functions. Table S7: List of all protein names abbreviated in Figure 4 with their functions. Table S8: List of all protein names abbreviated in Figure 5 with their functions.
