# Supplementary material for: Identification of Estrogen-Responsive Proteins in Mouse Seminal Vesicles Through Mass Spectrometry-Based Proteomics
Source: Pharmaceuticals (Basel). 2024 Nov 9;17(11):1508. doi: 10.3390/ph17111508 (PMC11597337; doi:10.3390/ph17111508)
Supplement: Supplementary file 1 [file pharmaceuticals-17-01508-s001.zip › pharmaceuticals-3260396_FigureS4.pdf]

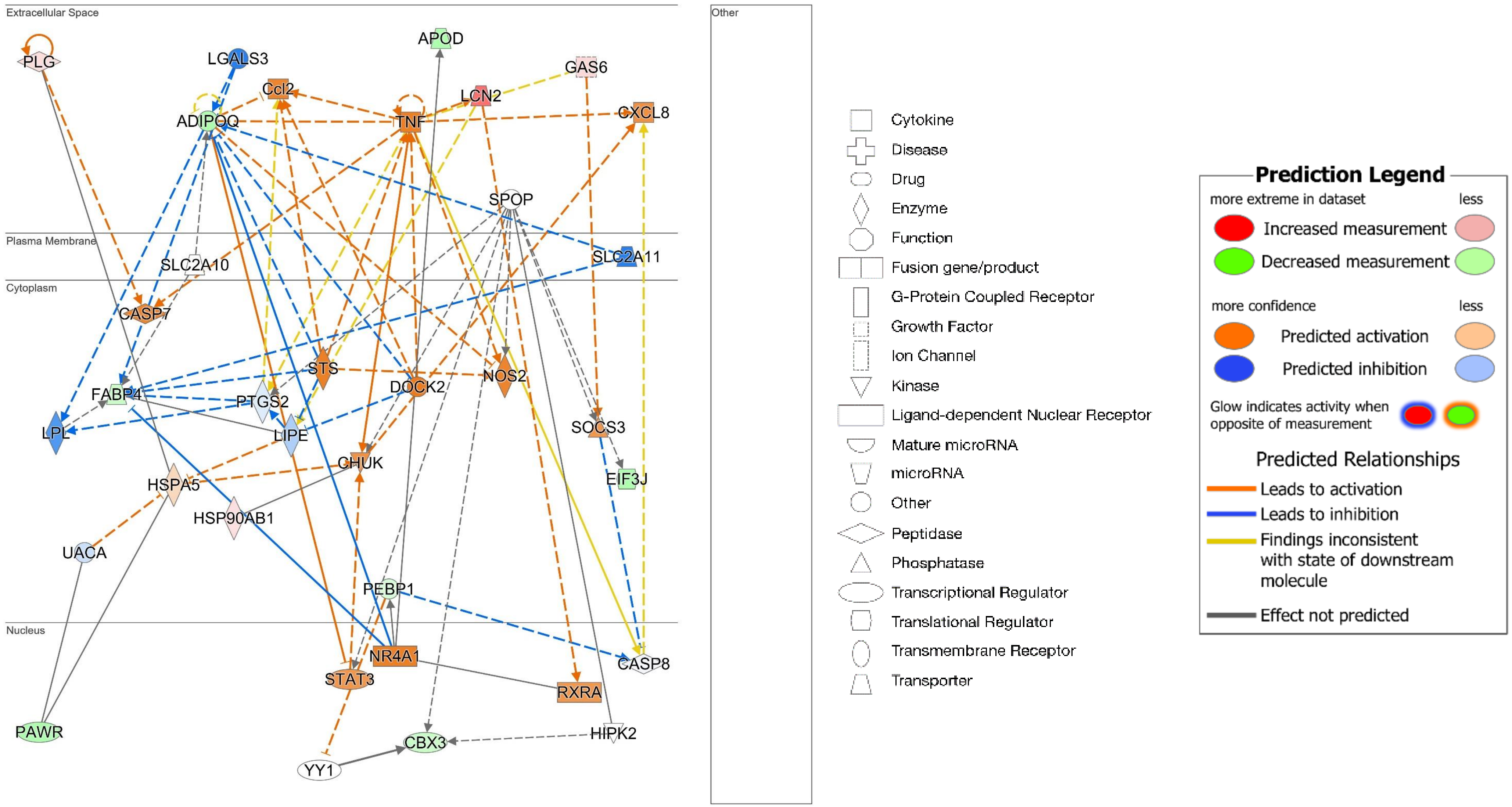

Figure S4: IPA® network linked to cellular movement, hematological system development and function, and immune cell trafficking.
