# Supplementary figures and images for: Identification of Estrogen-Responsive Proteins in Mouse Seminal Vesicles Through Mass Spectrometry-Based Proteomics
Source: Pharmaceuticals (Basel). 2024 Nov 9;17(11):1508. doi: 10.3390/ph17111508 (PMC11597337; doi:10.3390/ph17111508)

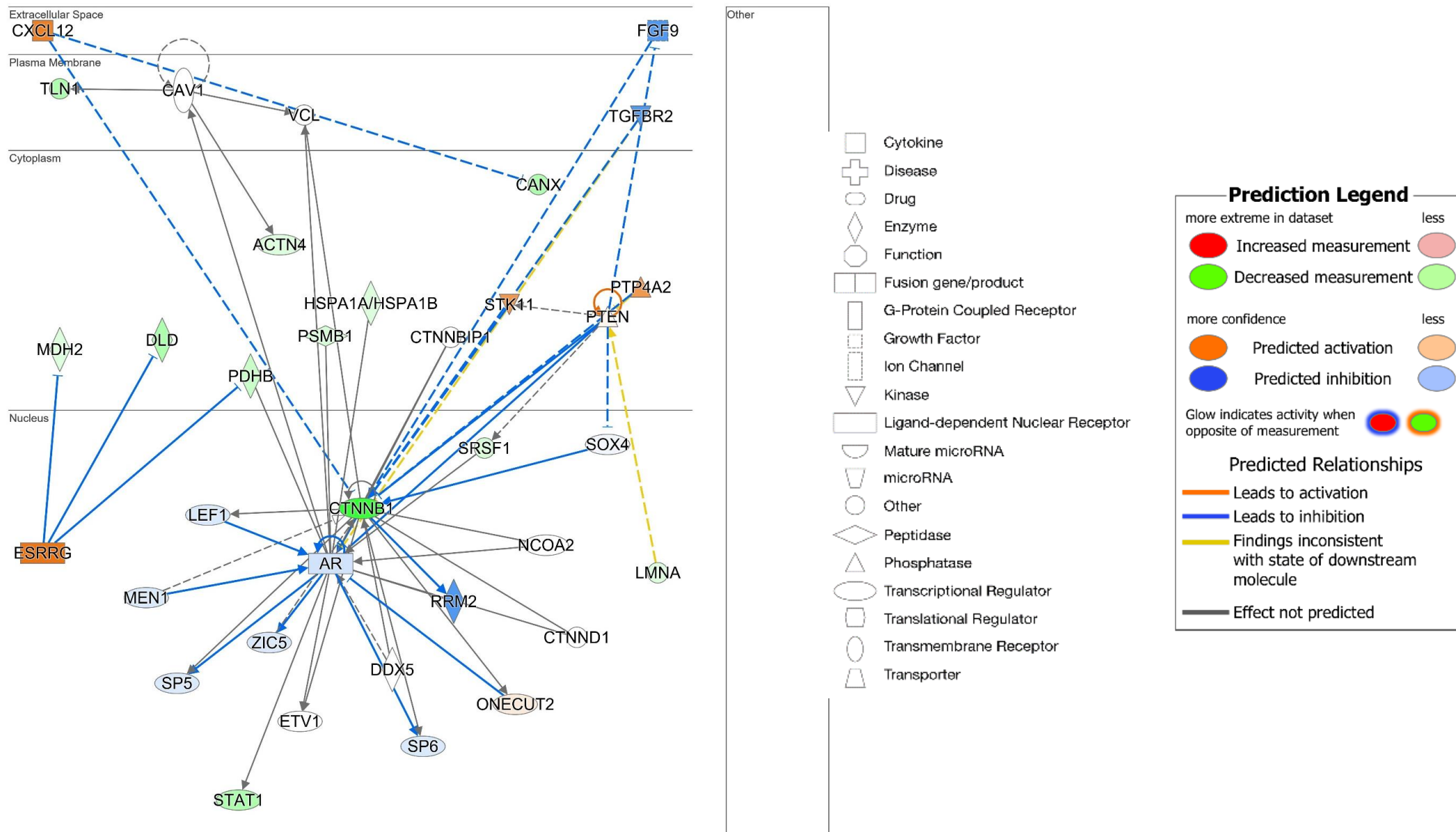

Figure S3: IPA® network linked to cancer, cellular movement, organismal injury and abnormalities.

Supplement: Supplementary file 1 [file pharmaceuticals-17-01508-s001.zip › pharmaceuticals-3260396_FigureS3.pdf]

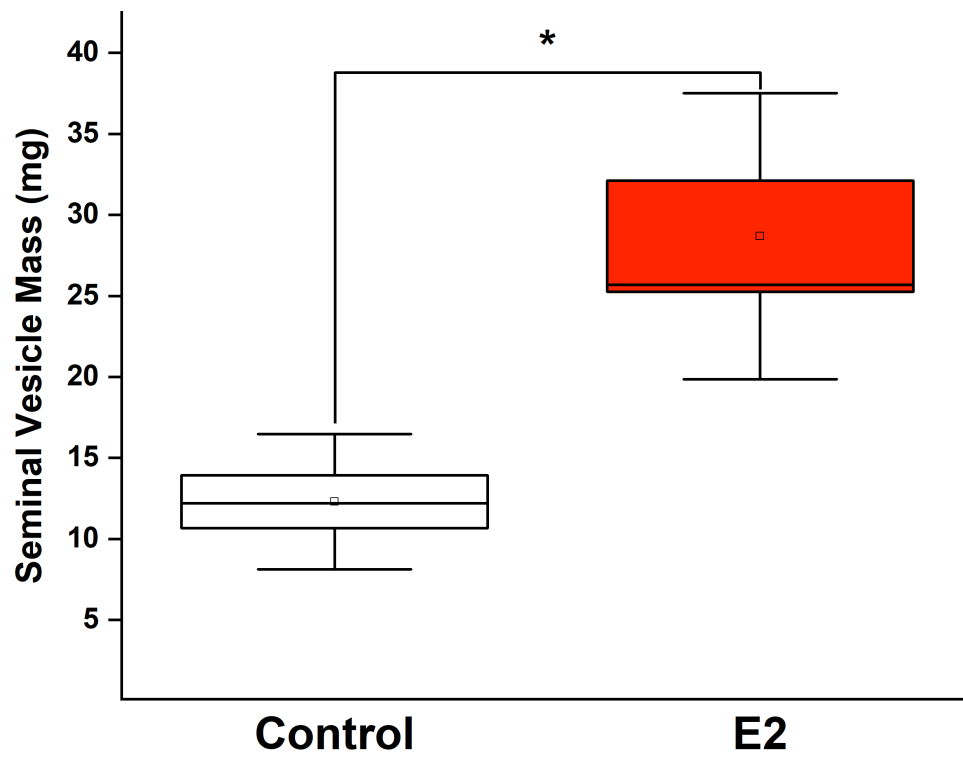

Figure S1. Seminal vesicle weight comparison between control and E2-treated animals.

Supplement: Supplementary file 1 [file pharmaceuticals-17-01508-s001.zip › pharmaceuticals-3260396_FigureS1.pdf]
